# ADRB3 induces mobilization and inhibits differentiation of both breast cancer cells and myeloid-derived suppressor cells

**DOI:** 10.1038/s41419-022-04603-4

**Published:** 2022-02-10

**Authors:** Zhiling Zhou, Jiaxin Zhan, Qiong Luo, Xinghua Hou, Shuxia Wang, Dingzhang Xiao, Zhi Xie, Haidan Liang, Shuguang Lin, Meng Zheng

**Affiliations:** 1 Zhuhai People’s Hospital, Zhuhai Hospital Affiliated with Jinan University, Zhuhai, China; 2 Guangdong Provincial Key Laboratory of Coronary Heart Disease Prevention, Guangdong Provincial People’s Hospital, Guangdong Academy of Medical Sciences, Guangzhou, China

**Keywords:** Breast cancer, Breast cancer

## Abstract

Metastatic tumors are mainly composed of neoplastic cells escaping from the primary tumor and inflammatory cells egressing from bone marrow. Cancer cell and inflammatory cell are remained in the state of immaturity during migration to distant organs. Here, we show that ADRB3 is crucial in cell mobilization and differentiation. Immunohistochemistry revealed ADRB3 expression is significantly more frequent in breast cancer tissues than in adjacent noncancerous tissues (92.1% vs. 31.5%). Expression of ADRB3 correlated with malignant degree, TNM stage and poor prognosis. Moreover, ADRB3 expression was markedly high in activated disseminated tumor cells, myeloid-derived suppressor cells (MDSCs), lymphocytes and neutrophil extracellular traps of patients. Importantly, ADRB3 promoted the expansion of MDSC through stimulation of bone marrow mobilization and inhibiting of the differentiation of immature myeloid cells. Furthermore, ADRB3 promoted MCF-7 cells proliferation and inhibited transdifferentiation into adipocyte-like cell by activating mTOR pathway. Ultimately, the MDSC-deficient phenotype of ADRB3 ^-/-^ PyMT mice was associated with impairment of mammary tumorigenesis and reduction in pulmonary metastasis. Collectively, ADRB3 promotes metastasis by inducing mobilization and inhibiting differentiation of both breast cancer cells and MDSCs.

## Background

Breast cancer (BC) is the most commonly occurring cancer in women [1, 2]. Metastasis is responsible for majority of deaths in patients who have been successfully treated, especially triple-negative breast cancer (TNBC) [3–5]. Metastatic tumors are mainly composed of neoplastic cells escaping from the primary tumor and inflammatory cells egressing from bone marrow. Cancer cell and inflammatory cell are remained in the state of immaturity during metastasis. Nevertheless, the mechanism of the upregulation of mobilization and the downregulation of differentiation of both cancer cells and inflammatory cells have not been well illustrated.

Of the various factors, dormant disseminated tumor cells (DTCs) reawakening and immunosuppression play pivotal roles in metastasis. Cell cycle exit is a common precondition for both reentry of DTCs into the dormant state and differentiation of the immune cells. Therefore, defects in cell cycle exit impair the reentry dormancy of DTCs as well as result in the accumulation of immature immune cells, which promote pre-metastatic niche (PMN) formation. Cell entry into the cell cycle is always associated with upregulation of the nucleolar function and increased nucleolar size [6]. The nucleolus is a multifunctional nuclear domain, which, in addition to its well-known role in ribosome biogenesis [7], plays crucial role in controlling cell cycle [8] and preventing early G1 phase from returning G0 phase linked to differentiation. Almost all cancer types display abnormalities in their morphology and number of nucleoli [9]. Indeed, cardinal changes occur in the proteome of the nucleolus during tumorigenesis and viral infection. Non-nucleolar proteins (NNPs) are directed to the nucleolus and vice versa [10]. Assembly of nucleolus in early G1 phase could depend on NNPs shuttling between the nucleoplasm and cytoplasm. Inhibition of nucleo-cytoplasmic shuttling of NNPs would disrupt the nucleolar assembly thereby induce cell cycle exit and differentiation. It is also becoming evident that mTORC1/4E-BP pathway, which regulates ribosome biogenesis, is often exploited by cancer cells to sustain proliferation [11].

The tumor microenvironment (TME) play role in the outgrowth of dormant cancer cells [12–14]. Once cancer cells avert immunosurveillance induced by inflammation in the TME, they emerge from dormancy and initiate colonization. Tumor-associated macrophages and neutrophils (TAMs and TANs) that descend from myeloid-derived suppressor cells (MDSCs) to facilitate BC immune evasion are the major components of the TME [15–18]. MDSCs are comprised of heterogenous immature myeloid cells (IMCs) that appear in cancer and in pathologic conditions associated with chronic inflammation or stress [19, 20]. Given MDSCs’ central role in BC promotion, inhibition of their expansion and promoting IMCs differentiation may attenuate BC. MDSCs expansion governed by the sympathetic nervous system (SNS) that control myelopoiesis and myeloid cell mobilization to meet the increased demand for myeloid cells. As a physiologic stress response, myelopoiesis is regulated by augmented activation of SNS through β3 adrenergic receptor (ADRB3) [21, 22]. However, the relationship of ADRB3 with MDSCs mobilization in BC has never been explored.

It is becoming increasingly clear that the SNS, PMN and MDSCs demonstrate cross-talk during DTCs reactivation by means of β-adrenoceptors (β1, β2, β3) pathways. Recent studies proved that upregulated activity of the SNS enhanced metastasis, which could be antagonized by β-blockers [23–25]. However, β1/β2 antagonist nadolol treatment increased number of lung metastases and splenic MDSC frequency in the mammary-specific polyomavirus middle T antigen overexpression mouse (MMTV-PyMT) [26]. Viral infection is often accompanied by temporal localization of NNPs in the nucleolus and participate in the infectious process [27–29]. We speculate that in MMTV-PyMT mice, ADRB3 signaling acts to activate DTCs by regulation of nucleolus functions. We previously demonstrated that ADRB3 promotes tumor cell proliferation and inflammation through activation monocyte-derived alveolar macrophages in non-small cell lung carcinoma. Anti-ADRB3 monoclonal antibody induced cell cycle arrest and accumulation of the tumor suppressor p53 [30], which involved in nucleolar stress [31]. The lung is also one of the most common places for metastatic BC. The recruitment of MDSCs promotes a pro-inflammatory and immunosuppressive PMN in the lung to sustain the formation of metastatic tumors. Here, we evaluated the role of ADRB3 in MDSCs mobilization, PMN formation, DTCs reactivation and BC metastasis.

## Materials and methods

### Reagents

β3-adrenoceptor agonist BRL 37344, antagonist SR59230A were from Sigma-Aldrich (St. Louis, MO). mTOR, p-CENP-A (S7), p-Rb (S780), Cyclin D1, p-Rictor (T1135), p-4E-BP1(T37/46), p-AKT (S473), p53, flag, PML, and Ki-67 antibody were from Cell Signaling (Danvers, MA). ADRB3, MPO, interleukin-6 (IL-6), Nucleolin, Fibrillarin, Neutrophil elastase (NE), IFN-γ, PD-L1, CD8, and CD68 antibody were from Abcam (Cambridge, MA). Transfections were performed using Lipofectamine 3000 (Invitrogen) for plasmid. The pcDNA3-Flag-ADRB3 was constructed by our lab.

### Human tissue samples

BC tissue microarrays and patient profiles were obtained from Shanghai Outdo Biotech. Peripheral blood samples and malignant pleural effusions were obtained from eight BC patients treated in Zhuhai People’s Hospital between 2019 and 2020. In addition, the blood samples of eight healthy individuals who received physical examination were used as the normal control group.

### Cell culture

Human BC cell line MCF-7 were purchased from ATCC (Manassas, USA). All cells were grown in DMEM (Gibco, USA) supplemented with 10% fetal bovine serum and 1% penicillin/streptomycin at 37 °C in a humidified air with 5% CO_2_.

### Generation of ADRB3-deficient MMTV-PyMT mouse model

MMTV-driven polyoma middle T antigen (PyMT) mice (FVB/N background) and ADRB3 knockout mice (FVB/N background) were purchased from Jackson Laboratory. PyMT mice were bred with ADRB3^−/−^ mice to generate PyMT/ADRB3^−/−^ mouse. All animals were housed and experimented in AAALAC accredited facility of Nanjing Biomedical Research Institute of Nanjing University (Nanjing, China). In spontaneous tumor formation experiments, tumor measurement started at week 6 after birth and continued until week 16 for WT and KO groups. Tumor sites were palpated biweekly to monitor tumor latency and progression in female PyMT/ADRB3^+/+^ or PyMT/ADRB3^−/−^ mice. Mice were euthanized until week 18, and the lungs were isolated for detecting the metastatic foci.

### Tumor implantation

Female Balb/c nude mice (4–5 weeks old) were purchased and maintained at the Laboratory Animal Center of Sun Yat-sen University. Tumor formation was induced in Balb/c nude mice by the injection of 10 ^6^ MCF-7 cells. The longer (*L*) and the width (*W*) of the tumor were measured every 3 days in period. The tumor volume was calculated via the formula *V* = (*LW*^2^)/2. Mice were divided randomly into two groups (10 animals/group) when the tumor volume reached 50–60 mm^3^. Mice were intravenously injected through the tail vein with 50 µg SR59230A every 3 days for 5 weeks, and the control group was injected with DMSO. All mice used in this study were maintained and used at the First Affilicated Hospital of Sun Yat-sen University mouse facility according to institutional guidelines and animal study proposals approved by the Institutional Animal Care and Use Committee.

### Bromo-deoxyuridine (BrdU) immunostaining

Seed MCF-7 cells at a density of 10^5^ cells/mL in culture plates and allow cells to grow for 24 h before BrdU labeling. BrdU was added to culture medium to a final concentration of 10 μm. After BrdU labeling for 2 h, medium was removed and fixed cells in 2% formaldehyde. 2 M HCl denatured DNA for 30 min. Immunostained with a monoclonal anti-BrdU antibody followed by fluorescent-dye conjugated secondary antibody.

### Cell-viability assays

Cell viability was determined using thiazolyl blue tetrazolium bromide (MTT) assays according to the protocols of the manufacturer and as previously described [30].

### Cell transdifferentiation

For the induction of adipocyte-like cell transdifferentiation, MCF-7 cells were seeded at a density of 5 × 10^4^ cells/mL/well in a 24-well plate. At confluence (day 0), the cultured preadipocytes were induced to differentiate by the addition of differentiating medium (DMEM containing 0.5 μM dexamethasone, 0.5 mM 3-isobutyl-1-methylxanthine, and 1 μg/mL insulin (DMI)) from day 0 to day 12. The medium was refreshed every 3 days. At day 12, differentiated MCF-7 cells were subjected to Oil red O solution. SR 59230 A was dissolved in dimethyl sulfoxide (DMSO) and was added to the cell culture medium at concentrations of 0.1 and 1 μM from day 0 to day 3, respectively.

### Oil-red O staining

Cells were fixed with 10% formaldehyde solution for 15 min. Fixed cells were washed with 60% isopropyl alcohol and were air dried. Stain with freshly prepared Oil red O working solution 15 min. After the removal of Oil red O solution, cells were washed four times with distilled water and imaged. Accumulated intracellular Oil red O dye was completely extracted by 1 mL of isopropanol and quantified by measuring its absorbance at 490 nm.

### Immunofluorescence and immunohistochemistry (IHC)

Cells were fixed in 4% formaldehyde and permeabilized in 0.2% Triton X-100. After incubation with primary antibody followed by secondary antibody in the dark, cell nuclei were stained by incubation with 4′,6-diamidino-2-phenylindole (DAPI). Images were acquired on confocal microscope (Leica, Germany) and analyzed with Fluorchem software (Alpha Innotech, USA). ADRB3 expression levels were expressed as the geometric mean fluorescence intensity (MFI). IHC assays were performed as previously described [30]. The percentage of immunostaining and the staining intensity (0, negative; 1+, weak; 2+, moderate; and 3+, strong) were recorded. *H*-score was calculated using the following formula: *H*‐score = (% of cells of weak × 1) + (% of cells of moderate × 2) + (% of cells of strong × 3).

### Western blot analysis

Protein was separated by sodium dodecyl sulfate-polyacrylamide gel electrophoresis and transferred to a polyvinylidene difluoride membrane. After blocking, immunodetection was performed using primary and horseradish peroxidase-labeled secondary antibodies, followed by detection with enhanced chemiluminescence.

### Statistical analyses

Results for continuous variables are presented as means ± SEM. Treatment groups were compared using independent sample *t*-tests. Pairwise multiple comparisons used one-way ANOVA (two-sided). *P*-value of <0.05 was considered statistically significant. All analyses were performed using IBM SPSS Statistics software version 23.0 (Chicago, USA).

## Results

### ADRB3 overexpression correlates with poorer tumor differentiation, advanced clinical stage, and shorter survival

**Fig. 1:**
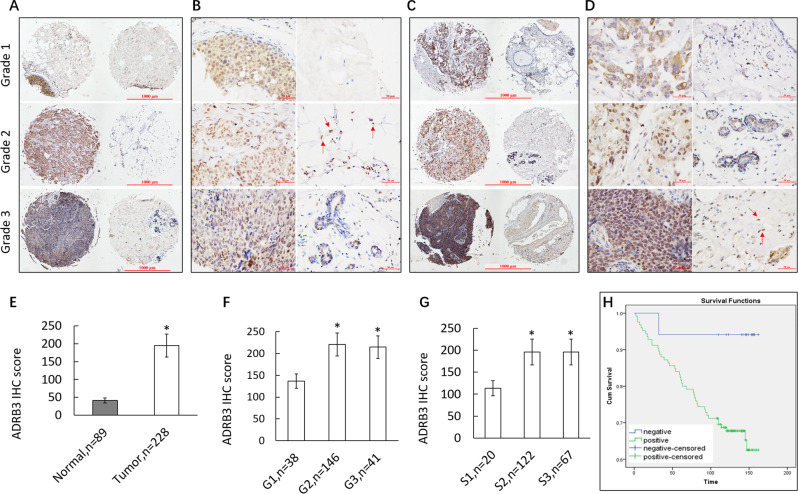
ADRB3 expression in breast cancer. **A**, **B** Representative tissue spots of ADRB3 expression in cancerous tissue and paracancerous tissue of different grade in non-TNBC. Each row represents two specimens from the same patient; the left is cancerous tissue, the right is paracancerous tissue, bars = 50 μm. Red arrows indicate macrophages. **C**, **D** Representative tissue spots of ADRB3 expression in cancerous tissue and paracancerous tissue of different grade in TNBC. Each row represents two specimens from the same patient; the left is cancerous tissue, the right is paracancerous tissue, bars = 50 μm. Red arrows indicate macrophages. **E** The ADRB3 staining score was significantly increased in cancerous tissues (194.2 ± 32.6) compared with adjacent normal tissues (41.7 ± 6.8), *P < 0.01. **F** Poorly differentiated BC samples showed higher ADRB3 protein expression, compared with those well-differentiated BC samples, *P < 0.01. **G** The ADRB3 staining score was significantly increased along with more advanced TNM stage in BC tissues, *P < 0.01. **H** Kaplan–Meier curves of the overall survival of 142 breast cancer patients with negative or positive ADRB3 expression. The ordinate is the cumulative survival rate, and the abscissa is the survival time (months).

Immunohistochemistry revealed ADRB3 expression is significantly more frequent in tumorous tissues than in adjacent noncancerous tissues (Table 1), and frequency of ADRB3 expression does not differ between non-TNBC (*n* = 203; Fig. 1A, B) and TNBC (*n* = 25; Fig. 1C, D). The positive rate of ADRB3 expression in cancer tissue was 92.1% (210/228), of which 56.7% (119/210) were intermediate and strongly positive. In paracancerous tissue, the positive rate of ADRB3 expression was 31.5% (28/89), of which 3.6% (1/28) were intermediate and strongly positive (Supplementary Table. 1). ADRB3 protein was primarily present in the cytoplasm and nucleus. The ADRB3 staining score was significantly increased in cancerous tissues (194.2 ± 32.6, *n* = 228) compared with adjacent normal tissues (41.7 ± 6.8, *n* = 89) (*P* < 0.01; Fig. 1E). Furthermore, high expression levels of ADRB3 were observed in tumor-infiltrating macrophages.

**Table 1:** Correlations between the expression of ADRB3 and histological grade, AJCC staging (6th) of BC.

	Patients (*n*)	ADRB3 positive (*n*)	ADRB3 positive rate (%)	
<55 years	129	114	88.3	*χ*^2^ = 5.7, *P* = 0.017.
≥55 years	99	96	97.0	
Cancer tissue	228	210	92.1	*χ*^2^ = 125.8, *P* < 0.001.
Paracancerous tissue	89	28	31.5	
TNBC	25	23	92.0	
NON-TNBC	203	187	92.1	
Grade				
1	38	28	73.7	
2	146	139	95.2	Compared with grade 1, *χ*^2^ = 16.7, *P* < 0.01.
3	41	40	97.6	Compared with grade 1, *χ*^2^ = 9.4, *P* < 0.01.
TNM stage				
1	20	13	65.0	
2	122	111	90.9	Compared with stage 1, *χ*^2^ = 10.5, *P* < 0.01.
3	67	67	100.0	Compared with stage 1, *χ*^2^ = 25.5, *P* < 0.01. Compared with stage 2, *χ*^2^ = 6.4, *P* = 0.011.

The expression rate of ADRB3 in cancer tissues was positively correlated with the malignant degree. The higher the malignant tumor grade, the stronger the expression of ADRB3. The positive rate in grade (G) 1, 2, 3 samples was 73.7% (28/38), 95.2% (139/146), 97.6% (40/41), respectively. The positive rate in G2 or G3 was significantly higher than in G1 (*P* < 0.01; Fig. 1F). The positive rate of ADRB3 in cancer tissues was also correlated with the TNM stage (S). The more advanced the disease, the stronger the expression of ADRB3. In S1, the positive rate was 65.0% (13/20), but in S3, the positive rate increased to 100% (67/67). The ADRB3 positive rates in S2 or S3 was significantly higher than that in S1 (*P* < 0.01; Fig. 1G).

The positive rate of ADRB3 in cancerous tissues of patients who were ≥55 years was significantly higher than in patients <55 years (97.0% vs. 88.3%, *P* = 0.017). ADRB3 expression in cancerous tissue was positively correlated with Ki-67 (Supplementary Table. 2-3). The ADRB3 expression level in cancer tissue was significantly correlated with Ki-67 expression (*r* = 0.296, *P* = 0.02). Ki-67-positive samples had a higher ADRB3 score than Ki-67-negative samples (*P* = 0.01).

Kaplan–Meier analysis for 142 patients with survival data showed that patients with ADRB3-negative had a significantly higher overall survival than patients with ADRB3-positive (*P* = 0.025, Supplementary Table. 4). The mean survival time in the two groups were 154 months and 126 months, respectively. Shown in the survival curve (Fig. 1H), with the increase in follow-up time, the survival rate in ADRB3-positive patients decreased gradually up to 170 months, when the survival rate was ~60%; in contrast, the survival rate in ADRB3-negative patients decreased more slowly and was still above 90% after 170 months.

### High ADRB3 expression in DTCs and immature immune cells of BC patients

**Fig. 2:**
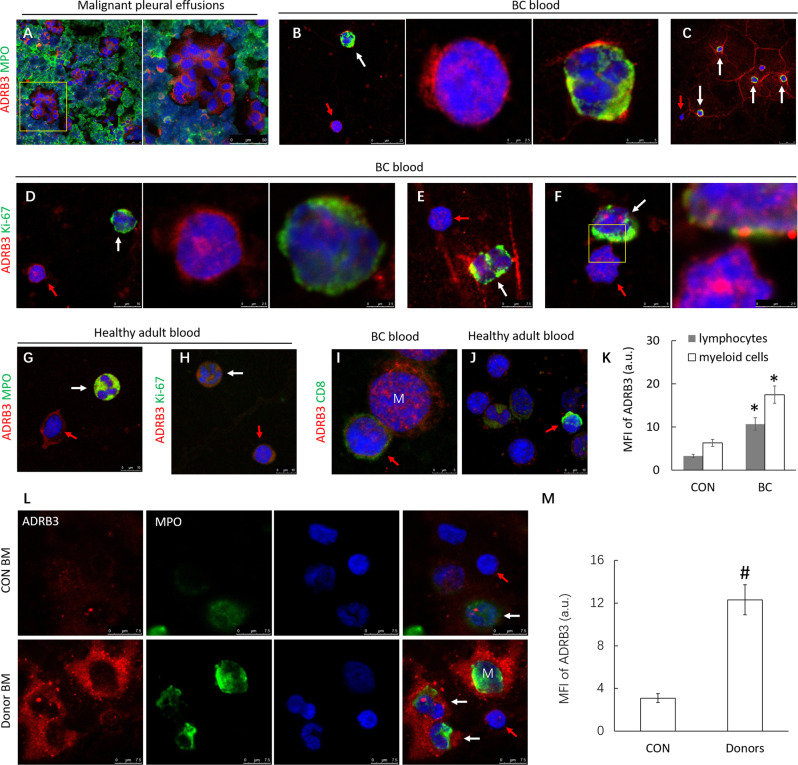
High ADRB3 expression in DTCs and IMCs of BC patients. **A** Representative images of immunofluorescence results for anti-ADRB3 (red) and anti-MPO (green) staining on MPE smear show that ADRB3 was expressed on myeloid cells (MPO^+^ cells). Confocal images were collected using a 63× oil-immersion lens. **B** Immunofluorescence of ADRB3 (red) and MPO (green) in blood smear of BC patients. Middle and right: Highly magnified view of left. White arrows indicate myeloid cells, red arrows indicate lymphocytes. **C** Immunofluorescence of ADRB3 (red) and MPO (green) on NETs in blood smear of BC patients. White arrows indicate myeloid cells, red arrows indicate lymphocytes. **D** Immunofluorescence of ADRB3 (red) and Ki-67 (green) in blood smear of BC patients. Middle and right: Highly magnified view of left. White arrows indicate myeloid cells, red arrows indicate lymphocytes. **E** Immunofluorescence of ADRB3 (red) and Ki-67 (green) on NETs in blood smear of BC patients. **F** Immunofluorescence of ADRB3 (red) and Ki-67 (green) on adherens junction between lymphocytes and neutrophils in blood smear of BC patients. White arrows indicate myeloid cells, red arrows indicate lymphocytes. **G**, **H** Immunofluorescence of ADRB3 (red), MPO (green), and Ki-67 (green) in blood smear of healthy controls. White arrows indicate myeloid cells, red arrows indicate lymphocytes. **I** Immunofluorescence of ADRB3 (red) and CD8 (green) in blood smear of BC patients. M: monocyte. Red arrows indicate lymphocytes. **J** Immunofluorescence of ADRB3 (red) and CD8 (green) in blood smear of healthy controls. Red arrows indicate lymphocytes. **K** Mean fluorescent intensities (MFI) of ADRB3. Data is expressed as means ± SEM of six independent experiments. *P < 0.05 compared with the healthy controls. **L**, **M** Immunofluorescence of ADRB3 (red) and MPO (green) in BMC of healthy person (*n* = 5) and hematopoietic stem cell transplantation donors (*n* = 5). ^#^*P* < 0.01 compared with the healthy adults.

Malignant pleural effusions (MPE) are rich source of DTCs, we thus examined the expression of the ADRB3 in DTCs from MPE. Consistent with previous reports that ADRB3 is highly upregulated on myeloid-derived cells [30], the MPE smears of BC patients (*n* = 8) shows high expression ADRB3 in cytoplasm, nucleoplasm and nucleolus of DTCs that surrounded by ADRB3^+^ MPO^+^ myeloid cell (Fig. 2A), indicating adhesive ADRB3^+^ IMCs provide a protective effect on activated DTCs survival. We thus hypothesize that DTCs which had spread to the distant organs can create a defensive shield consisting of ADRB3^+^ myeloid cell, which allows evasion of immune surveillance and therefore facilitates reactivation of dormant DTCs.

In addition, we found that ADRB3^+^ Ki-67^+^ immature neutrophil with its nucleus in an elongated “band” shape accumulated in peripheral blood from patients (Fig. 2B, D). These cells released ADRB3-rich neutrophil extracellular traps (NETs) that can entangle and perhaps suppress cytotoxic T cell function (Fig. 2C, E). It was revealed that serum NETs were identified as a predictive marker for the onset of liver metastases in BC patients [32]. We detected numerous ADRB3-rich NETs in peripheral blood from patients, suggesting that cancer cells can induce NETs formation and neutrophil recruitment via ADRB3. In contrast, plasma NETs were absent in healthy controls. Lymphocytes that adhered to ADRB3^+^ myeloid cells or NETs also highly expressed ADRB3, especially in the adhesive area (Fig. 2F). Both lymphocytes and myeloid cells from BC patients had significantly higher level of ADRB3 compared with healthy adults (average age <30 years) (Fig. 2G, H). Consistent with increased ADRB3, we observed that ADRB3 relocated to the nucleus and nucleolus in both neutrophils and lymphocytes of BC patients. The nuclear translocation of ADRB3 could contribute to chromatin decondensation and formation of NETs. In contrast, there were very few ADRB3 in the nucleus of immune cells from healthy donors. When the evaluation was extended to T cells, we observed a significantly higher expression of ADRB3 on CD8^+^ T cells in peripheral blood from BC patient (Fig. 2I) compared with healthy controls (Fig. 2J).

BC patients showed increased MFI of ADRB3 expression in lymphocytes and myeloid cells compared to healthy controls (10.5 ± 1.6 vs. 3.3 ± 0.5; *P* < 0.01; 17.5 ± 2.8 vs. 6.3 ± 0.9; *P* < 0.05, respectively) (Fig. 2K). BC patient lymphocytes were ADRB3^high^ Ki-67^-^, which arrested in early G1 phase, whereas ADRB3^high^ Ki-67^+^ MDSCs with highly replicative potential lead to excessive inflammation and form a shied that protects DTC from immune effector cells.

To evaluate if ADRB3 expression is also upregulated during myeloid development, we detected the level of ADRB3 in bone marrow cells (BMC) of hematopoietic stem cells (HSCs) donors (*n* = 5) injected Granocyte (recombinant glycosylated human granulocyte colony-stimulating factor), which is widely used to stimulate granulopoiesis and mobilize HSCs into the blood. We found that there was a significant increase of ADRB3 expression in myeloid cells of donor’s BM after treatment of Granocyte (Fig. 2L, M). ADRB3 expression was quickly decreased and returned to normal levels after the drug was stopped. Thus, ADRB3 protein expression transiently increases and then rapidly decreases during the early stages of myeloid progenitor development, suggesting that ADRB3 could promote the transient expansion of IMCs through stimulation of IMCs mobilization and inhibiting the differentiation of IMCs.

### ADRB3 prevents cell cycle exit linked to differentiation by facilitating nucleolar assembly and activating mTOR

**Fig. 3:**
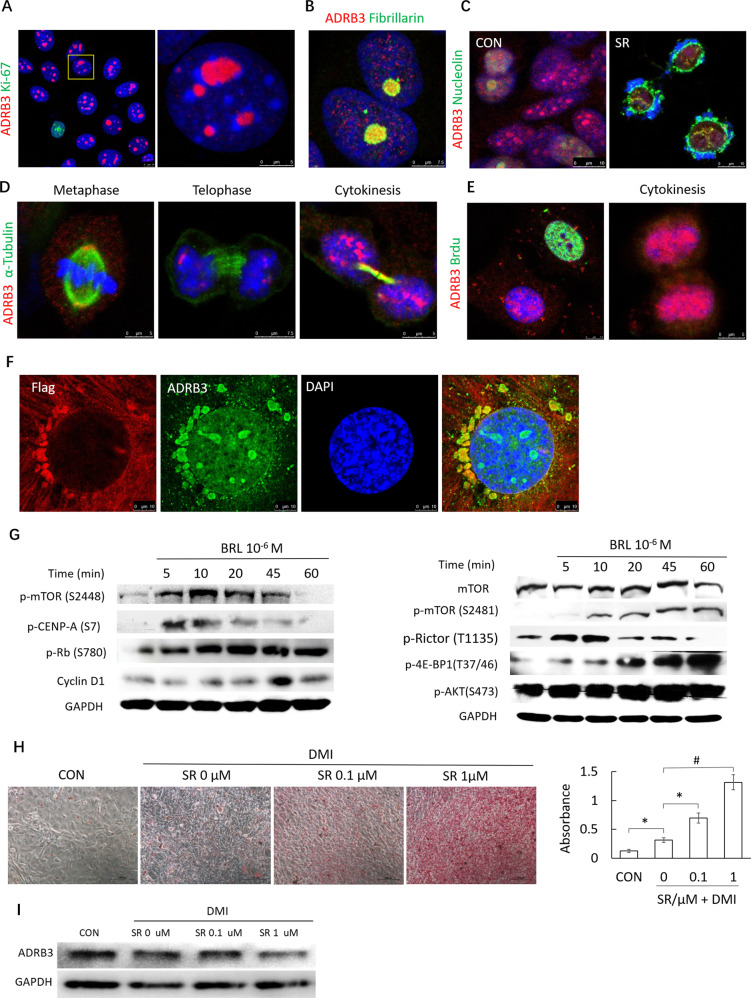
ADRB3 translocates into the nucleolus during early G1 phase and activates mTOR/4E-BP pathway. **A** Intracellular localization of ADRB3 (red) and Ki-67 in MCF-7 cells. DAPI was used for costaining nuclei (blue). Confocal images were collected using a 63× oil-immersion lens. **B** Intracellular localization of ADRB3 (red) and fibrillarin in MCF-7 cells. **C** Intracellular localization of ADRB3 (red) and nucleolin in MCF-7 cells treated with or without 10 µM SR59230A (SR) for 24 h. **D** Intracellular localization of ADRB3 (red) and α-tubulin in MCF-7 cells during mitosis. **E** ADRB3 was enriched in the nucleolus of G1 or cytokinesis cells which were unable to incorporate BrdU. **F** Immunofluorescence of ADRB3 (green) and flag-tagged ADRB3 (red) in MCF-7 cells transfected pcDNA3-FLAG-ADRB3. **G** Representative images of immunoblot showing p-mTOR (S2448), p-CENP-A (S7), p-Rb (S780), cyclin D1, p-mTOR (S2481), p-Rictor (T1135), p-4E-BP1(T37/46), p-AKT (S473), and GAPDH levels in MCF-7 cells treated with 10 μM BRL37344 (BRL). **H** Intracellular lipid was stained by Oil Red O (ORO) on day 12 and quantification of ORO dye by spectorphotometer at 490 nm. All experiments were repeated at least three times. *P < 0.05, ^#^*P* < 0.01. **I** Representative images of immunoblot showing ADRB3 and GAPDH levels in MCF-7 cells treated with or without SR59230A.

ADRB3 accumulated in the nucleolus of Ki-67^-^ MCF-7 cells, which were in early G1 phase (Fig. 3A). ADRB3 colocalized with either dense fibrillar component (DFC) protein fibrillarin (Fig. 3B) or granular component (GC) protein such as Ki-67 and nucleolin (Fig. 3C). The colocalization of proteins in the same structure does not indicate whether or not these proteins interact. However, the nuclear translocation of nucleolin was impaired by ADRB3 antagonist SR59230A (SR) (Fig. 3C). The assembly of the nucleolus starts during telophase [33]. ADRB3 localized dynamically to the centrosome during metaphase and to the nucleolus during telophase and cytokinesis (Fig. 3D). ADRB3 was enriched within the nucleolus of G1 phase cells that were unable to incorporate BrdU, as well as the cells during cytokinesis (Fig. 3E), indicating ADRB3 participated in nucleolar assembly from cytokinesis to G1 phase. Flag-tagged ADRB3 colocalized with the anti-ADRB3 (Abcam) (Fig. 3F), indicating the ADRB3 antibody staining was specific. Further, cytoplasm flag-tagged ADRB3 was unable to transport across the nuclear membrane along microtubules, suggesting that ADRB3 translocated from the cytoplasm to nucleolus.

Next, we assessed the effect of ADRB3 on MCF-7 cells growth in vitro. MTT assays showed that SR significantly decreased cell viability. The 72-h inhibitory concentration 50 (IC50) of SR in the MCF-7 cells were found to be 3.95 μg/mL. Compared with the control group, SR significantly increased the proportion of the MCF-7 cells in G1 phase (67.3 ± 12.1% vs. 28.0 ± 8.4%, *P* < 0.05).

The mammalian target of rapamycin (mTOR), in response to the energy state and nutrient status, plays a role in the coordination of cell growth and the cell cycle [34, 35]. We then analyzed the effect of ADRB3 on activity of mTORC1 and mTORC2. The phosphorylation of p-mTOR S2448 (mTORC1) induced by ADRB3 agonist BRL37344 (BRL) peaked at 10 min and p-mTOR S2481(mTORC2) did at 60 min (Fig. 3G). Furthermore, p-CENP-A (S7), p-Rb (S780), p-Rictor (T1135), p-4E-BP1(T37/46) and p-AKT (S473) were increased by BRL in a time-dependent manner (Fig. 3G).

Cancer cells are undifferentiated because they cannot exit the cell cycle. Cancer cells may share characteristics with 3T3-L1 preadipocytes, which undergo growth arrest before adipocyte differentiation. Therefore, we investigated whether ADRB3 affected the transdifferentiation of MCF-7 cells into adipocyte-like cells by impairing cell cycle exit. Transdifferentiation was initiated by adding SR (0.1 or 1 μM) in MCF-7 cells before being exposed to differentiation media containing DMI. SR-treatment promoted their differentiation into adipocytes-like cells in a dose-dependent manner as measured by Oil Red O staining (Fig. 3H) associated with decreased ADRB3 expression (Fig. 3I).

### SR59230A inhibits breast cancer xenograft tumor growth

**Fig. 4:**
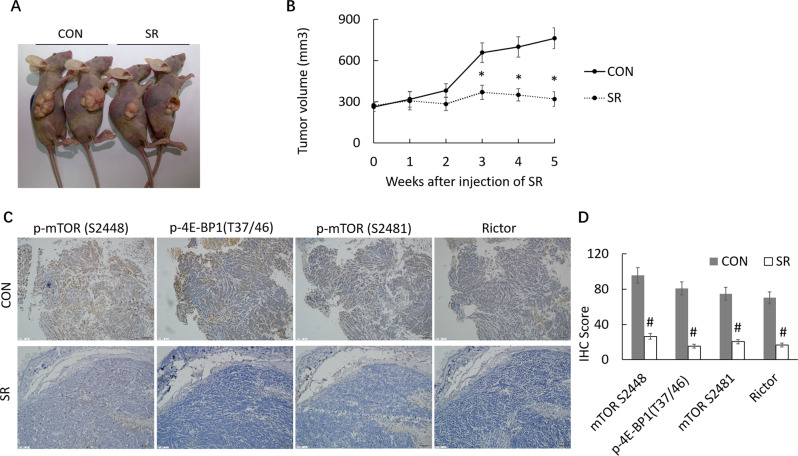
Effects of SR59230A on mouse xenograft tumors in vivo. **A** Representative images of nude mice and tumors. **B** Growth curve of tumors for phosphate-buffered saline or SR59230A treatment group. *n* = 10 in each group. *P < 0.05 compared with the controls. **C**, **D** Immunohistochemical analysis of p-mTOR S2448, p-4E-BP1(T37/46), p-mTOR S2481 and rictor expression in xenografts (×200 magnification), ^#^*P* < 0.01 compared with the controls.

To confirm the growth effect of ADRB3 in vivo, we performed an in vivo tumorigenesis study by inoculating MCF-7 into nude mice with or without SR59230A treatment. Mice were intravenously injected through the tail vein with 50 µg SR59230A every 3 days. The results showed that SR59230A led to slower growth rate and smaller tumor volume (321.3 ± 51.4 mm^3^) compared with the control group (762.7 ± 74.8 mm^3^; *P* < 0.05) (Fig. 4A, B). The expression of p-mTOR S2448, p-4E-BP1(T37/46), p-mTOR S2481 and Rictor were decreased in tumor of SR group (Fig. 4C, D).

### ADRB3 loss impairs MMTV-PyMT tumor formation and metastasis

**Fig. 5:**
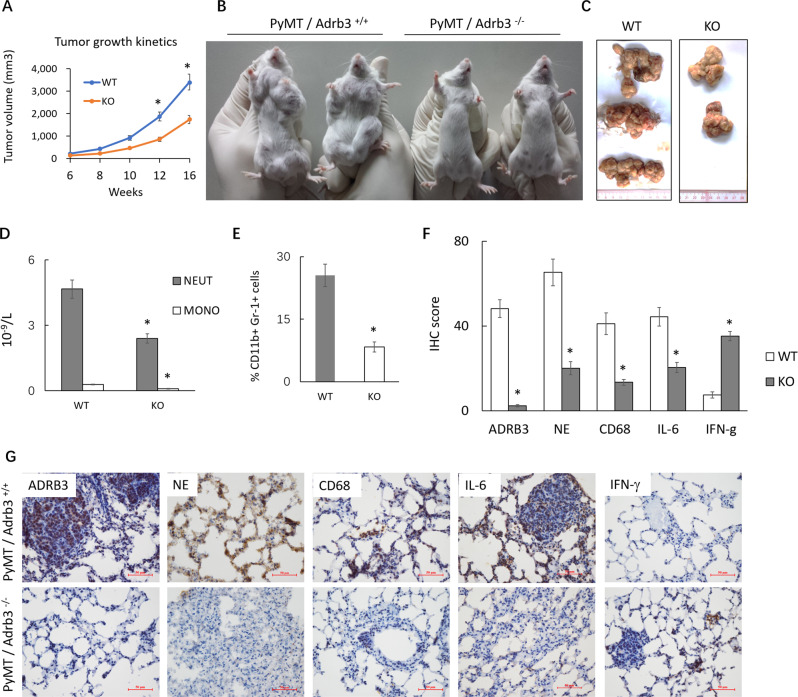
ADRB3 loss impairs MMTV-PyMT tumor formation and metastasis. **A** Tumor growth kinetics (*n* = 10 WT, *n* = 10 KO mice, 3 tumors per mouse), *P < 0.05. Two-way ANOVA test with Holm-Šidák multiple-comparisons test. **B** Observation of breast tumor of 18-week-old PyMT-ADRB3^+/+^ and PyMT-ADRB3^-/-^ mice. **C** The photograph of the excised tumors. **D** Numbers of neutrophils, monocytes and lymphocytes in the blood of 18-week-old PyMT-ADRB3^+/+^ and PyMT-ADRB3^-/-^ mice. EDTA anti-coagulated blood samples were used to obtain a complete blood count with automated hematology analyzer. **E** Frequencies of CD11b^+^ Gr-1^+^ cells in spleens from PyMT-ADRB3^+/+^ and PyMT-ADRB3^-/-^ mice, *P < 0.05. **F**, **G** Immunohistochemical analysis of ADRB3, neutrophil elastase (NE), CD68, IL-6, and IFN-γ and Rictor expression in lung tissues (×200 magnification), *P < 0.05 compared with PyMT-ADRB3^+/+^ mice.

MMTV-PyMT mice develop highly invasive mammary tumors that metastasize spontaneously to the lung [36–39]. PyMT, the main polyoma virus (Py) oncogene, by itself will only transform these cells when p53 is inactivated [40]. To evaluate the relevance of ADRB3 gene in promotion of tumor growth in immune competent mice, we crossed ADRB3^-/-^ mice with the MMTV-PyMT transgenic mice, a mouse model of breast cancer that mirrors the multistep progression of human breast cancers, and generated female cohorts with the genotypes of PyMT-ADRB3^+/+^ mice and PyMT-ADRB3^-/-^ mice. In female PyMT-ADRB3^+/+^ mice (*n* = 10), mammary tumors occurred as early as week 6, and by week 10, 80% of the mice developed tumors. In contrast, we observed a 2–3 week delay in palpable tumors in female PyMT-ADRB3^-/-^ mice (*n* = 10), and 50% of the mice developing palpable tumors after week 10. By 18 weeks of age, whereas all PyMT-ADRB3^+/+^ mice developed multiple tumors, reduced incidence of tumor formation (100% vs. 70%, *P* = 0.003) and dramatic reduction in tumor volume were found in PyMT-ADRB3^-/-^ mice (3403 ± 321 vs. 1,743 ± 150 mm^3^, *P* < 0.05) (Fig. 5A–C).

PyMT-ADRB3^-/-^ mice developed markedly reduced incidence of lung metastases compared with PyMT-ADRB3^+/+^ mice (100% vs. 60%, *P* = 0.001). Significantly reduced the number of neutrophils and monocytes was detected in PyMT-ADRB3^-/-^ mice as compared with control littermates (4.7 ± 0.4 vs. 2.4 ± 0.2, *P* < 0.05; 4.7 ± 0.4 vs. 2.4 ± 0.2, *P* < 0.05) (Fig. 5D), suggesting that cancers, through a systemic effect on the host by ADRB3, can induce an increase in peripheral blood neutrophils, which are predisposed to NET formation. Frequencies of CD11b^+^ Gr-1^+^ MDSCs were decreased in the spleens of PyMT-ADRB3^-/-^ mice (25.3 ± 2.7% vs. 8.2 ± 1.2%; *P* < 0.05, Fig. 5E). Immune cells shape the microenvironment around the metastatic foci and are important modulators of the metastatic cascade [41]. To determine whether deletion of ADRB3 affects PMN for the malignant tumor cells, we analyzed the expression of several genes related to inflammation, macrophages and T cells by IHC using the specimens of mice lung tissues (Fig. 5F, G). In the PyMT-ADRB3^-/-^ mice, the lung tissues exhibited significantly lower expression levels of neutrophil elastase (NE) and interleukin-6 (IL-6) (*P* < 0.05). NE is termed as NET core signature [42]. In contrast to macrophages, neutrophils were not considered to be major players in the TME because they have a short life span. However, ADRB3-induced NETs could exist for a long time and contribute to the formation of PMN, which shielded DTCs from clearance of cytotoxic T lymphocytes. The number of CD68^+^ macrophages in the lung parenchyma was significantly decreased upon ADRB3 deletion. Furthermore, the expression of interferon gamma (IFN-γ), a representative Th1 cytokine, in the lung of PyMT-ADRB3^-/-^ mice was importantly higher than that of the PyMT-ADRB3^+/+^ mice. These results show that inhibiting the recruitment of MDSCs into the pre-metastatic lung, through ADRB3 depletion, maintain cytotoxic T-cell function and keep dormant DTC from growing.

## Discussion

In the present study, using tissue microarrays and ADRB3 knockout MMTV-PyMT mice models, we report that ADRB3 expression correlates with BC aggressiveness, and leads to BC progression, metastasis, and immunosuppression by preventing either cancer cells or IMCs from exiting cell cycle. In addition, we highlight that ADRB3 expression is not only restricted to cancer cells, but it is also expressed in activated DTCs, proliferative MDSCs and activated T cells. Particularly, we demonstrated that ADRB3 can (i) reactivate dormant BC cells, (ii) prevent the transdifferentiation of MCF-7 cells into adipocyte-like cells, (iii) regulate nucleolar assembly and (iv) promote the expansion of MDSC through stimulation of myelopoiesis and inhibiting of the differentiation of myeloid cells. Moreover, ADRB3 activation in MDSCs induces pro-inflammatory cytokines secretion, NETs formation and recruitment of MDSCs into the pre-metastatic lung to form a shied that protects DTC. Noteworthy, frequency of ADRB3 expression does not differ between non-TNBC and TNBC. Therefore, we propose that the ADRB3 is a major tumor driver and a vulnerability in TNBC.

It has become increasingly clear that successful cancer immunotherapy will be possible only with a strategy that involves the elimination of suppressive factors from the body. As MDSCs are one of the main immunosuppressive factors in cancer, suppressing these cells function or reducing their numbers could support the immune system’s response to tumors. ADRB3 deletion results in substantial decrease in the presence of MDSCs probably through inhibiting the expansion of MDSCs from bone marrow progenitors or promoting their differentiation into mature myeloid cells in PyMT mice. In conditions of acute stress, there is a transient expansion of IMCs population through ADRB3 pathway, which then quickly differentiates into mature myeloid cells accompanied by degradation of ADRB3. However, cancer cells chronically induce ADRB3 expression in IMCs as important ‘gatekeepers’ that prevent cell cycle exit linked terminal differentiation. Future studies will reveal whether ADRB3 can be considered part of a myeloid cell regulatory network.

Cellular proteins that are not normally present in the nucleolus can be relocated to the nucleolus and participate in the cancer process and viral infection. During viral infection numerous viral components localize in nucleoli, while non-nucleolar proteins reach the nucleolus [27]. ADRB3 loss impedes mammary tumor occurrence through the interplay between viral proteins (PyMT) and nucleolus of mammary epithelial cells in MMTV-PyMT mice.

Ribosome biogenesis is a limiting factor for dormant cell growth. Abnormal increases in nucleolar size and number caused by dysregulation of ribosome biogenesis have emerged as hallmarks in the majority of cancers [43]. ADRB3 accumulated in the nucleolus of Ki-67-negative MCF-7 cells, suggesting that the achievement of an adequate ribosome regulated by ADRB3 during the dormant phase makes it possible for the cell to overcome the G0-G1 phase restriction point. A key player for the regulation of ribosomal synthesis in response to extracellular conditions is the mTOR [44]. We found that ADRB3 increased the activation of mTOR, which suggests that ADRB3 may function to coordinate mTOR-dependent regulation of ribosomal synthesis.

T-cell senescence and exhaustion induced by malignant tumors are two important dysfunctional states that coexist in cancer patients [45]. Under normal conditions, naive T cells are actively maintained in a quiescent state that is an important protective mechanism against senescence. However, cancer cells may induce naïve T cell to arrest in G1 phase and subsequent senescence by awakening quiescence through upregulation of ADRB3 without antigen stimulation. Thus, persistent delivery of costimulatory signals via ADRB3, as may occur during tumorigenesis or viral infections, can exhaust the naïve T-cell pool and is sufficient to induce lethal immunodeficiency. Moreover, β3-adrenoceptors have been reported to increase with aging [46]. Our findings also show that the positive rate of ADRB3 in cancerous tissues of patients who were ≥55 years was significantly higher than in patients <55 years (Table 1), suggesting ADRB3 is associated with cellular senescence. T-cell exhaustion is a state of T-cell dysfunction that arises during viral infections and cancer [47], and the ADRB3 signaling might play a role in this process. Exhausted effector T cells are unable to release IFN-γ [48]. Deficiency of ADRB3 upregulates IFN-γ expression in the lungs of MMTV-PyMT mice, suggesting that blocking the ADRB3 pathway restores T cell function.

## Conclusions

In conclusion, this study has identified ADRB3 as an important regulatory factor in BC progression and metastasis, possibly acting through MDSCs. Consequently, therapeutics targeted at ADRB3 could potentially benefit patients.

## Supplementary information

Reproducibility checklist

Supplementary Table 1

Supplementary Table 2

Supplementary Table 3

Supplementary Table 4

## Data Availability

The datasets used and analyzed during the current study are available from the corresponding author on reasonable request.

## References

[1] Torre LA, Islami F, Siegel RL, Ward EM, Jemal A (2017). Global cancer in women: burden and trends. Cancer Epidemiol Biomark Prev.

[2] Bray F, Ferlay J, Soerjomataram I, Siegel RL, Torre LA, Jemal A (2018). Global cancer statistics 2018: GLOBOCAN estimates of incidence and mortality worldwide for 36 cancers in 185 countries. CA Cancer J Clin.

[3] O’Shaughnessy J (2005). Extending survival with chemotherapy in metastatic breast cancer. Oncologist.

[4] Guarneri V, Dieci MV, Conte P (2013). Relapsed triple-negative breast cancer: challenges and treatment strategies. Drugs..

[5] Kast K, Link T, Friedrich K, Petzold A, Niedostatek A, Schoffer O (2015). Impact of breast cancer subtypes and patterns of metastasis on outcome. Breast Cancer Res Treat.

[6] Derenzini M, Montanaro L, Treré D (2009). What the nucleolus says to a tumour pathologist. Histopathology..

[7] Volarevic S, Stewart MJ, Ledermann B, Zilberman F, Terracciano L, Montini E (2000). Proliferation, but not growth, blocked by conditional deletion of 40S ribosomal protein S6. Science.

[8] Tsai RY, Pederson T (2014). Connecting the nucleolus to the cell cycle and human disease. FASEB J.

[9] Derenzini M, Trerè D, Pession A, Montanaro L, Sirri V, Ochs RL (1998). Nucleolar function and size in cancer cells. Am J Pathol.

[10] Weeks SE, Metge BJ, Samant RS (2019). The nucleolus: a central response hub for the stressors that drive cancer progression. Cell Mol Life Sci.

[11] Saxton RA, Sabatini DM (2017). mTOR signaling in growth, metabolism, and disease. Cell..

[12] Barkan D, Green JE, Chambers AF (2010). Extracellular matrix: a gatekeeper in the transition from dormancy to metastatic growth. Eur J Cancer.

[13] Kaushik S, Pickup MW, Weaver VM (2016). From transformation to metastasis: deconstructing the extracellular matrix in breast cancer. Cancer Metastasis Rev.

[14] Lu P, Weaver VM, Werb Z (2012). The extracellular matrix: a dynamic niche in cancer progression. J Cell Biol.

[15] Cha YJ, Koo JS (2020). Role of tumor-associated myeloid cells in breast cancer. Cells.

[16] Hajizadeh F, Aghebati Maleki L, Alexander M, Mikhailova MV, Masjedi A, Ahmadpour M (2021). Tumor-associated neutrophils as new players in immunosuppressive process of the tumor microenvironment in breast cancer. Life Sci.

[17] Laoui D, Movahedi K, Van Overmeire E, Van den Bossche J, Schouppe E, Mommer C (2011). Tumor-associated macrophages in breast cancer: distinct subsets, distinct functions. Int J Dev Biol.

[18] Shou D, Wen L, Song Z, Yin J, Sun Q, Gong W (2016). Suppressive role of myeloid-derived suppressor cells (MDSCs) in the microenvironment of breast cancer and targeted immunotherapies. Oncotarget..

[19] Gabrilovich DI (2017). Myeloid-derived suppressor cells. Cancer Immunol Res.

[20] Millrud CR, Bergenfelz C, Leandersson K (2017). On the origin of myeloid-derived suppressor cells. Oncotarget.

[21] Katayama Y, Battista M, Kao WM, Hidalgo A, Peired AJ, Thomas SA (2006). Signals from the sympathetic nervous system regulate hematopoietic stem cell egress from bone marrow. Cell..

[22] Dutta P, Courties G, Wei Y, Leuschner F, Gorbatov R, Robbins CS (2012). Myocardial infarction accelerates atherosclerosis. Nature..

[23] Zhang D, Ma QY, Hu HT, Zhang M (2010). β2-adrenergic antagonists suppress pancreatic cancer cell invasion by inhibiting CREB, NFκB and AP-1. Cancer Biol Ther.

[24] Partecke LI, Speerforck S, Käding A, Seubert F, Kühn S, Lorenz E (2016). Chronic stress increases experimental pancreatic cancer growth, reduces survival and can be antagonised by beta-adrenergic receptor blockade. Pancreatology..

[25] Cole SW, Sood AK (2012). Molecular pathways: beta-adrenergic signaling in cancer. Clin Cancer Res.

[26] Dawes RP, Burke KA, Byun DK, Xu Z, Stastka P, Chan L (2020). Chronic stress exposure suppresses mammary tumor growth and reduces circulating exosome TGF-β content via β-adrenergic receptor signaling in MMTV-PyMT mice. Breast Cancer (Auckl).

[27] Salvetti A, Greco A (2014). Viruses and the nucleolus: the fatal attraction. Biochim Biophys Acta.

[28] Iarovaia OV, Ioudinkova ES, Velichko AK, Razin SV (2021). Manipulation of cellular processes via nucleolus hijaking in the course of viral infection in mammals. Cells.

[29] Emmott E, Hiscox JA (2009). Nucleolar targeting: the hub of the matter. EMBO Rep.

[30] Zheng M, Zhou Z, Tian X, Xiao D, Hou X, Xie Z (2020). ADRB3 expression in tumor cells is a poor prognostic factor and promotes proliferation in non-small cell lung carcinoma. Cancer Immunol Immunother.

[31] Rubbi CP, Milner J (2003). Disruption of the nucleolus mediates stabilization of p53 in response to DNA damage and other stresses. EMBO J.

[32] Yang L, Liu Q, Zhang X, Liu X, Zhou B, Chen J (2020). DNA of neutrophil extracellular traps promotes cancer metastasis via CCDC25. Nature..

[33] Muro E, Gébrane-Younís J, Jobart-Malfait A, Louvet E, Roussel P, Hernandez-Verdun D (2010). The traffic of proteins between nucleolar organizer regions and prenucleolar bodies governs the assembly of the nucleolus at exit of mitosis. Nucleus..

[34] Cuyàs E, Corominas-Faja B, Joven J, Menendez JA (2014). Cell cycle regulation by the nutrient-sensing mammalian target of rapamycin (mTOR) pathway. Methods Mol Biol.

[35] Fingar DC, Richardson CJ, Tee AR, Cheatham L, Tsou C, Blenis J (2004). mTOR controls cell cycle progression through its cell growth effectors S6K1 and 4E-BP1/eukaryotic translation initiation factor 4E. Mol Cell Biol.

[36] Fantozzi A, Christofori G (2006). Mouse models of breast cancer metastasis. Breast Cancer Res.

[37] Guy CT, Cardiff RD, Muller WJ (1992). Induction of mammary tumors by expression of polyomavirus middle T oncogene: a transgenic mouse model for metastatic disease. Mol Cell Biol.

[38] Attalla S, Taifour T, Bui T, Muller W (2021). Insights from transgenic mouse models of PyMT-induced breast cancer: recapitulating human breast cancer progression in vivo. Oncogene..

[39] Lin EY, Jones JG, Li P, Zhu L, Whitney KD, Muller WJ (2003). Progression to malignancy in the polyoma middle T oncoprotein mouse breast cancer model provides a reliable model for human diseases. Am J Pathol.

[40] Lomax M, Fried M (2001). Polyoma virus disrupts ARF signaling to p53. Oncogene..

[41] Kitamura T, Qian BZ, Pollard JW (2015). Immune cell promotion of metastasis. Nat Rev Immunol.

[42] Dwyer M, Shan Q, D’Ortona S, Maurer R, Mitchell R, Olesen H (2014). Cystic fibrosis sputum DNA has NETosis characteristics and neutrophil extracellular trap release is regulated by macrophage migration-inhibitory factor. J Innate Immun.

[43] Pelletier J, Thomas G, Volarević S (2018). Ribosome biogenesis in cancer: new players and therapeutic avenues. Nat Rev Cancer.

[44] Xiao L, Grove A (2009). Coordination of ribosomal protein and ribosomal RNA gene expression in response to TOR signaling. Curr Genomics.

[45] Zhao Y, Shao Q, Peng G (2020). Exhaustion and senescence: two crucial dysfunctional states of T cells in the tumor microenvironment. Cell Mol Immunol.

[46] Birenbaum A, Tesse A, Loyer X, Michelet P, Andriantsitohaina R, Heymes C (2008). Involvement of beta 3-adrenoceptor in altered beta-adrenergic response in senescent heart: role of nitric oxide synthase 1-derived nitric oxide. Anesthesiology..

[47] Blank CU, Haining WN, Held W, Hogan PG, Kallies A, Lugli E (2019). Defining ‘T cell exhaustion’. Nat Rev Immunol.

[48] Yi JS, Cox MA, Zajac AJ (2010). T-cell exhaustion: characteristics, causes and conversion. Immunology..

