# Supplementary material for: ADRB3 induces mobilization and inhibits differentiation of both breast cancer cells and myeloid-derived suppressor cells
Source: Cell Death Dis. 2022 Feb 10;13(2):141. doi: 10.1038/s41419-022-04603-4 (PMC8831559; doi:10.1038/s41419-022-04603-4)
Supplement: Supplementary file 2 — Supplementary Table 1 [file 41419_2022_4603_MOESM2_ESM.docx]

| Supplementary Table 1. The expression of ADRB3 in cancer and paracancerous tissues | | | | |  | | |
| --- | --- | --- | --- | --- | --- | --- | --- |
| ADRB3 expression | - | + | ++ | +++ | | Sum |  |
| Cancerous tissue（n） | 18 | 91 | 80 | 39 | | 228 |  |
| Rate | 7.9% （18/228） | 39.9%（91/228） | 35.1%（80/228） | 17.1%（39/228） | |  |  |
| Paracancerous tissue（n） | 61 | 27 | 1 | 0 | | 89 |  |
| Rate | 68.5%（61/89） | 30.3%（27/89） | 1.1%（1/89） | 0.0% | |  |  |
