# Supplementary material for: ADRB3 induces mobilization and inhibits differentiation of both breast cancer cells and myeloid-derived suppressor cells
Source: Cell Death Dis. 2022 Feb 10;13(2):141. doi: 10.1038/s41419-022-04603-4 (PMC8831559; doi:10.1038/s41419-022-04603-4)
Supplement: Supplementary file 3 — Supplementary Table 2 [file 41419_2022_4603_MOESM3_ESM.docx]

Supplementary Table 2. Spearman's rank correlation coefficient of ADRB3 and clinical parameters

|  | Her 2 | Ki-67 | P53 |
| --- | --- | --- | --- |
| Spearman's rank correlation coefficient | 0.167 | 0.296^*^ | 0.145 |

*P<0.05
