# Supplementary material for: ADRB3 induces mobilization and inhibits differentiation of both breast cancer cells and myeloid-derived suppressor cells
Source: Cell Death Dis. 2022 Feb 10;13(2):141. doi: 10.1038/s41419-022-04603-4 (PMC8831559; doi:10.1038/s41419-022-04603-4)
Supplement: Supplementary file 4 — Supplementary Table 3 [file 41419_2022_4603_MOESM4_ESM.docx]

Supplementary Table 3. Ki-67 positive samples had a higher ADRB3 score than Ki-67 negative samples

| Ki-67 | -(n=6) | +(n=99) | ++/+++(n=99) |
| --- | --- | --- | --- |
| ADRB3 score | 51±8 | 185±23^*^ | 274±32^#^ |

Compared with Ki67-negative samples, *P=0.01; compared with Ki67 weakly positive (+) samples, #P=0.04.
