# Supplementary material for: ADRB3 induces mobilization and inhibits differentiation of both breast cancer cells and myeloid-derived suppressor cells
Source: Cell Death Dis. 2022 Feb 10;13(2):141. doi: 10.1038/s41419-022-04603-4 (PMC8831559; doi:10.1038/s41419-022-04603-4)
Supplement: Supplementary file 5 — Supplementary Table 4 [file 41419_2022_4603_MOESM5_ESM.docx]

| Supplementary Table 4. Case processing summary | | | | |
| --- | --- | --- | --- | --- |
| Group | Total N | N of Events | Censored | |
|  |  |  | N | Percent |
| ADRB3 negative | 17 | 1 | 16 | 94.1% |
| ADRB3 positive | 125 | 42 | 83 | 66.4% |
| Overall | 142 | 43 | 99 | 69.7% |
